# Tumor microenvironment reprogramming combined with immunogenic enhancement by nanoemulsions potentiates immunotherapy

**DOI:** 10.1186/s12951-024-02401-y

**Published:** 2024-04-05

**Authors:** Wenqi Shen, Yecheng Li, Ziyi Yang, Wenjing Li, Yi Cao, Yilin Liu, Zheng Wang, Renjun Pei, Chungen Xing

**Affiliations:** 1 Department of General Surgery, Second Affiliated Hospital of Soochow University, Suzhou, 215004 P. R. China; 2 Affiliated Hospital of Xuzhou Medical University, Xuzhou, 221002 Jiangsu China; 3 CAS Key Laboratory for Nano-Bio Interface, Suzhou Institute of Nano-Tech and Nano-Bionics, Chinese Academy of Sciences, Suzhou, 215123 P. R. China; 4 School of Intelligent Finance and Business, Entrepreneur College, Xi’an Jiaotong-Liverpool University, Suzhou, 215123 P. R. China

**Keywords:** Cancer-associated fibroblasts, Tumor microenvironment, Intratumoral penetration, Colon cancer, Tumor immunotherapy

## Abstract

The combination of immune checkpoint inhibitors and immunogenic cell death (ICD) inducers has become a promising strategy for the treatment of various cancers. However, its efficacy remains unmet because of the dense stroma and defective vasculatures in the tumor microenvironment (TME) that restricts the intratumoral infiltration of cytotoxic T lymphocytes (CTLs). Herein, cancer-associated fibroblasts (CAFs)-targeted nanoemulsions are tailored to combine the ICD induction and the TME reprogramming to sensitize checkpoint blockade immunotherapy. Melittin, as an ICD inducer and an antifibrotic agent, is efficiently encapsulated into the nanoemulsion accompanied by a nitric oxide donor to improve its bioavailability and tumor targeting. The nanoemulsions exhibited dual functionality by directly inducing direct cancer cell death and enhancing the tumoral immunogenicity, while also synergistically reprogramming the TME through reversing the activated CAFs, decreasing collagen deposition and restoring tumor vessels. Consequently, these nanemulsions successfully facilitated the CTLs infiltration and suppressing the recruitment of immunosuppressive cells. A combination of AE-MGNPs and anti-CTLA-4 antibody greatly elicited a striking level of antitumor T-cell response to suppress tumor growth in CAFs-rich colorectal tumor models. Our work emphasized the integration of the ICD induction with simultaneous modulation of the TME to enhance the sensitivity of patients to checkpoint blockade immunotherapy.

**Supplementary Information:**

The online version contains supplementary material available at 10.1186/s12951-024-02401-y.

## Highlights

A synergistic therapy was developed by co-delivery of melittin and GSNO (AE-MGNPs) to amplify immunotherapy via remodeling tumor immunosuppressive microenvironment.

AE-MGNPs reprogrammed the activated CAFs, normalized the tumor vessels and induced immunogenic cell death to improve the CTLs infiltration while suppressing the recruitment of immunosuppressive cells.

A combination of AE-MGNPs and anti-CTLA-4 antibody greatly elicited a striking level of antitumor T-cell response to suppress tumor growth in CAFs-rich colorectal tumor models.

**Supplementary Information:**

The online version contains supplementary material available at 10.1186/s12951-024-02401-y.

## Introduction

Checkpoint blockade immunotherapy, which targets regulatory the pathway of T cells to unleash antitumor T cell responses, has been considered a revolutionary treatment against various types of malignancies. However, the relatively low immune response rates hinder the scope of its clinical application [1, 2]. Substantial evidences have shown that checkpoint blockade immunotherapy is only applicable to tumors with pre-existing of T-cells [3]. Fortunately, partial cancer treatments, such as radiotherapy, chemotherapy and hyperthermia therapy, have been found to modulate the initial cancer immunity to strengthen the efficacy of checkpoint inhibitors by triggering immunogenic cell death (ICD) including the generation of tumor antigens, exposure of damage-associated molecular patterns (DAMPs), and secretion of pro-inflammatory cytokine [4–6]. Although promising, the combination of an ICD strategy with checkpoint blockade immunotherapy remains unsatisfactory in activating antitumor immune responses.

Successful immunotherapy requires multiple key steps involving tumor antigen capture and presentation, effector T cell activation and expansion, direct cell-cell contact between immune effector cells and tumor cells, and the production of inflammatory cytokines to exert antitumor functions [7]. However, the immunosuppressive tumor microenvironment (TME) restricts the infiltration and activation of immune cells, and promotes further immunosuppression [8]. Among various cell types and extracellular components within the TME, cancer-associated fibroblasts (CAFs) have emerged as central players to shape the TME to an immunosuppressive phenotype by producing dense extracellular matrix (ECM) and secreting suppressive cytokines, effectively hindering the accumulation of T cells in the vicinity of cancer cells [9, 10]. In addition to CAFs, aberrant tumor vasculatures also counteract immunotherapy due to the inadequate blood delivery and reduced transmigration of lymphocytes and impairs antitumor immune responses by favoring immunosuppressive cells over immunostimulatory cells [11, 12].

Considering these features, a plausible strategy to sensitize checkpoint blockade immunotherapy is the induction of ICD, and its combination with TME modulation to overcome the obstacle of excessive ECM and defective vasculatures. Our previous studies, along with the work of other researchers have consistently demonstrated that eliminating CAFs can effectively reduce ECM deposition in the TME [13, 14]. However, recent reports have indicated that the direct elimination of CAFs increases the risk of tumor metastasis [15]. To mitigate potential risks, an alternative strategy that holds promise, is the reversion of activated CAFs into quiescent states, rather than depleting them directly [16, 17]. Melittin, a major component of Iranian honey bee (Apis mellifera) venom, has strong lytic activity against cell membranes and is a potent anti-cancer peptide that promotes the ICD of cancer cells [18]. More importantly, melittin exhibits therapeutic potential against fibrotic diseases by virtue of ability to inactivate fibrosis; however, studies on its efficacy on CAFs regulation are rare [19]. Therefore, we hypothesize that melittin plays a multifaceted role in cancer immunotherapy including the induction of ICD and the transformation of the activated CAFs into quiescent cells. However, clinical application of melittin in cancer treatment is restricted due to its non-specific hemolysis and rapid clearance [20]. To circumvent these drawbacks, drug delivery systems, such as, nanoparticles (NP) or polymer-peptide conjugates, have been proposed as potential solutions to mitigate these challenges [18]. Accordingly, reprogramming of CAFs can induce the inactivated state of CAFs and reduce ECM production to remodel the TME [21], which to some extent improves CD8^+^ T cell infiltration into tumor tissue. However, single modality therapy against CAFs showed unsatisfactory therapeutic efficacy because the aberrant tumor vasculature is also capable of impeding tumor infiltration of CTLs, which generate a protective barrier in tumor immunotherapy [22].

**Scheme 1:**
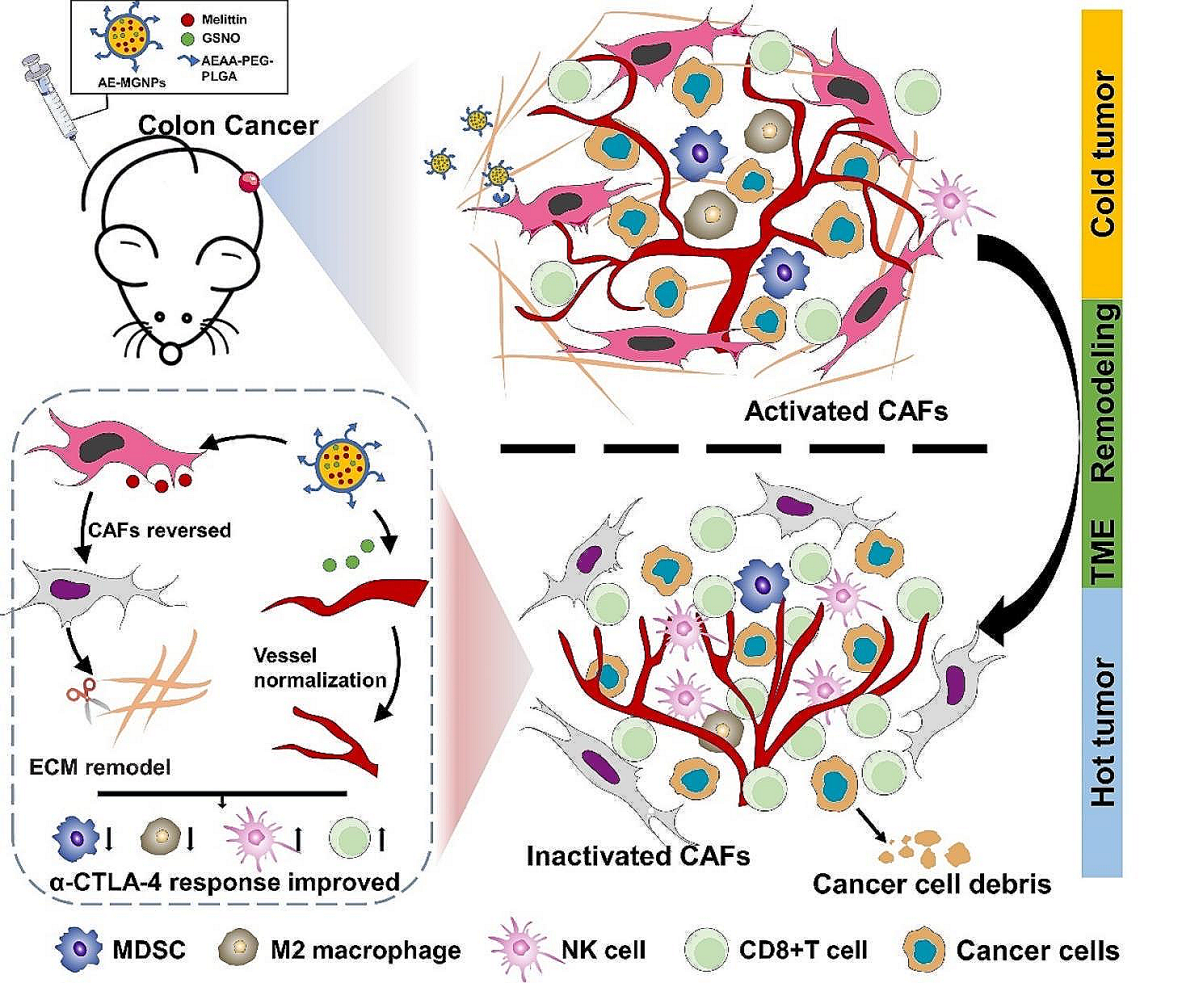
Nanomulsions remodel tumor immunosuppressive microenvironment through reversing the activated CAFs, normalizing the tumor vessels and inducing immunogenic cell death to potentiate immunotherapy

Nitric oxide (NO) has been reported to mediate vascular normalization and maintenance of vascular stabilization, thereby contributing to the normalization of tumor vessels [22–25]. S-nitroso glutathione (GSNO), as the endogenous NO donors to generate NO for tumor vascular normalization, is limited by its short half-life, poor tumor targeting and rapid release NO gas [26]. In this study, aminoethyl anisamide (AEAA)-modified poly (lactic-co glycolic acid) (PLGA) nanoemulsions were exploited to facilitate the delivery of melittin and GSNO (AE-MGNPs), delay their systemic elimination, improve their targeting functionality in the TME and prolong the NO release (Scheme 1). The prepared AE-MGNPs showed a selective cytotoxic activity against cancer cells, sparing CAFs and vascular endothelial cells. Furthermore, these nanoparticles induced and triggered ICD effect at the tumor site thereby promoting the activation and recruitment of immune cells. Notably, the AE-MGNPs reprogrammed the immunosuppressive TME by reversing the activated CAFs, decreasing collagen deposition and normalizing the tumor vessels, thus improving the infiltration of cytotoxic T lymphocytes and decreasing the frequency of immunosuppressive cells. Significantly, the co-administration of AE-MGNPs and anti-CTLA-4 antibody (α-CTLA-4) resulted in impressive tumor regression in CAFs-rich colorectal tumor models. These findings underscore the promising role of AE-MGNPs in augmenting the effectiveness of checkpoint blockade immunotherapy.

## Experimental section

### Materials

PLGA (15,000) (lactide-glycolide ratio: 50:50)-PEG (3400)-AEAA was purchased in Yusi Medicine Co., Ltd.(Chongqing, China). Poly (vinyl alcohol) (PVA, 30–70 kDa) and PLGA (Mw 7–17 k, lactide-glycolide ratio: 50:50) were acquired in Sigma Aldrich (Saint. Louis, USA). Melittin and S-nitrosoglutathione (GSNO) were acquired in Aladdin (Shanghai, China). Dialkylcarbocyanines (DiR and DiI) were acquired in Yeasen Biotechnology (Shanghai, China). FITC-labelled Lycopersicon esculentum lectin was gained in Vector Laboratories. Pimonidazole and Hypoxyprobe-redAPC-labelled antibodies were obtained from Hypoxyprobe (Massachusetts, USA). The in vivo anti-mouse CTLA-4 (CD152) antibody was obtained from BioXCell (New Hampshire, USA). Anti-Smooth Muscle Actin antibody was purchased in Santa Cruz Biotechnology (Texas, USA). Fibroblast Activation Protein (FAP) Rabbit Polyclonal Antibody was obtained from Beyotime (Shanghai, China).

### Preparation of AE-MGNPs

AE-MGNPs were obtained based on the solvent evaporation method (W_1_/O/W_2_ ) [27]. In short, 7.5 mg PLGA plus 2.5 mg PLGA-PEG-AEAA were mixed together and dissolved with 500 µL dichloromethane (DCM) as organic phase; after this, 3 mg melittin and 0.25 mg GSNO were dissolved in 50 µL ddH_2_O as the internal aqueous phase. A primitive water/oil emulsion was formed with a probe sonicator for 2 min (2 s on / 2 s off; 20% power) in ice. Subsequently, the internal emulsion (W_1_/O) was added to another 2% PVA (3 mL) dropwise and sonicated for 5 min (2 s on / 2 s off; 20% power) to form a double emulsion (W_1_/O/W_2_). DCM in the final emulsion was eliminated using a rotary evaporator. After undergoing three washes with ddH_2_O, AE-MGNPs were assembled through centrifugation (13,523 g, 20 min, 4 °C) and resuspended in ddH_2_O. AE-MNPs and AE-GNPs were synthesized accordingly using the same protocol by adding 3 mg melittin or 0.25 mg GSNO into the inter aqueous phase, while for synthesizing DiR or DiI-labelled AE-NPs, 100 µg of the respective dye was put in the organic phase.

### Characterization of the nanoemulsions

The zeta potential and average size of the obtained nanoemulsions were detected using dynamic light scattering (DLS, Malvern, UK). Morphological monitoring of the AE-MGNPs was conducted through transmission electron microscopy (TEM, Hitachi, Japan).

The quantity of melittin remaining in the AE-MGNPs wash supernatant was detected via the BCA protein concentration detection kit to ensure the encapsulation efficiency (EE%) and drug loading content (DL%) of melittin. The amount of GSNO in the NPs was determined as said by the quantity of free GSNO in the filtrate detected with the UV spectrum at 336 nm.

In vitro drug release of free GSNO and AE-GNPs was assessed. Briefly, 10 mg of the prepared AE-GNPs was suspended in 5 mL PBS solution (pH 7.4), which was divided into 10 groups on average, under gentle shaking at 37 °C (100 rpm). Each of the NPs suspensions was collected at predetermined time intervals through centrifugation, and 200 µL supernatant was assembled to quantify the NO_2_^−^ using the Griess assay. The concentration of NO_2_^−^ was used to calculate the release of NO from GSNO. Free GSNO was used as a control. Free GSNO was equally suspended in PBS under gentle shaking (100 rpm/min) at 37 °C, after which the specimens were assembled and quantified for three times at the same time point.

### Hemolysis assays

Fresh blood from healthy individuals was assembled using anticoagulant tubes containing EDTA. Red blood cells (RBCs) were isolated through centrifuging at 2000 rpm for 5 min. Next, isolated RBCs were purified using PBS for three times, and adjusted to the concentration of 5.0×10^7^/mL for the subsequent hemolysis assays. Then, free melittin and melittin-NPs (AE-MNPs) in different concentrations in 800 µL PBS were cultured with 200 µL of RBCs for 3 h in cell incubator. The supernatants of each group were assembled and the absorbance was detected through a microplate reader at 540 nm. RBC treated with ddH_2_O served as positive controls.

### Preparation and identification of cancer-associated fibroblasts

To trigger CAF differentiation, mouse embryonic fibroblast cell line NIH/3T3 was incubated together with TGF-β1 for 48 h. Briefly, 1 × 10^6^ NIH/3T3 cells (seeded in a 10^− cm^ plate for 24 h) were treated with TGF-β1 (at a dose of 10 ng/mL) for 48 h. For identification of CAFs, fibroblast activation protein-α (FAP-α) and α-smooth muscle actin (α-SMA), two kinds of CAFs’ specific biomarkers, were detected by confocal laser scanning microscopy (CLSM) and quantitative real time PCR (qPCR).

### Cytotoxicity test

Cell viability assay was detected via the Cell Counting Kit-8 (CCK-8) kit (Beyotime, Shanghai, China) to estimate the biocompatibility of the free AE-NPs. Briefly, CRC cell lines CT26, HUVECs, or CAFs were cultured in 96-well plates overnight. Fresh medium containing free AE-NPs were added to each well and treated for another 72 h. Following this, 10% CCK-8 solution was prepared and the cell viability was detected through documenting the absorbance using a microplate reader at 450 nm.

### Cellular uptake

CAFs and CT26 cells were used in the detection of the cellular uptake of nanoparticals. Briefly, 5 × 10^5^ CAFs and CT26 cells were cultivated in 6-well plates. Then, DiI-NPs and AE-DiI-NPs suspended in 2 mL of fresh medium were exchanged to the plates and cultured for 1 h. Then, washing the cells with PBS twice to remove free NPs. Trapped NPs in cells were quantitated via flow cytometry (BD Biosciences, New Jersey, USA). In addition, CLSM was also used to observe the targeting ability of AE-NPs in CAFs and CT26 cells. The CAFs and CT26 cells were treated with DiI-NPs and AE-DiI-NPs. Then, 5 µM Hoechst was used to stain the nucleus for CLSM analysis.

### Toxicity analysis

The toxicity of AE-MNPs and AE-GNPs to CT26 cells, CAFs, and HUVECs was investigated through CCK-8 assay. Briefly, 5000 CT26 cells, CAFs, and HUVECs were cultured overnight and incubated with fresh medium including free melittin, free GSNO, AE-MNPs, and AE-GNPs (at a dose of melittin 4 µg/mL, GSNO 30 µg/mL) in the well. Subsequently, 10% CCK-8 kit was added after 24 h treatment, and cell viability was according to the OD value at 450 nm via a microplate reader after 2 h incubation.

### ICD assay

CT26 cells were incubated with free melittin, free GSNO, AE-MNPs, AE-GNPs, or AE-MGNPs. The cells were stained using FITC-labelled anti-CRT and monitored via flow cytometry to analyze the expression of calreticulin (CRT) on the cell surface.

### Tumor environment modulation in vitro

CAFs reprogramming assay: 5 × 10^5^ CAFs were incubated with free melittin, AE-MNPs as well as AE-MGNPs for 24 h. qPCR and confocal immunofluorescence assay were conducted to assess the expression levels of FAP-α and α-SMA in CAFs cells.

Vascular function regulation assay: 5 × 10^5^ HUVEC cells were treated with free GSNO, AE-GNPs, or AE-MGNPs for another 24 h. qPCR assay was implemented to assess the expression level of angiogenesis-related genes, including ANGPT1, S1PR1, ANGPT2, VEGFA, and EGF, in the HUVECs.

### Tumor environment modulation in vivo

Female BALB/c mice were got in Skorui Biotechnology Co. LTD (Nanjing, China) (3–5 weeks, 18–22 g). A total of 4 × 10^6^ cells (CAFs/CT26 cells 1:3) were suspended using 100 µL PBS and then inoculated into the right flank of mice for the establishment of subcutaneous tumor model. Mice were then administered five injections containing PBS, AE-NPs, AE-MNPs, AE-GNPs, and AE-MGNPs (melittin 5 mg/kg, GSNO 0.5 mg/kg) intravenously every day. Tumors were resected to make sections for immunohistochemistry (IHC) analysis and Masson’s trichrome staining. Primary anti-α-SMA (ab5694; Abcam, Cambridge, UK) were used for IHC assay. For the analysis of tumor vascular maturity, tumor sections were stained with NG2 (55027-1-AP, Proteintech, Chicago, USA) and CD31 (ab28364, Abcam, Cambridge, UK).

For evaluation of tumor hypoxia degree, 60 mg/kg pimonidazole (Hypoxyprobe, Massachusetts, USA) were injected intravenously (iv) 1 h before mice were sacrificed. The presence of hypoxia in tissue sections was assessed through immunostaining with pimonidazole. An anti-Hypoxyprobe-APC-labelled antibody was utilized for the detection of hypoxic regions. All tumor sections were imaged with CLSM.

5 min before the mice were sacrificed, 100 µL of FITC-labelled Lycopersicon esculentum lectin (Vector Laboratories, iv, San Francisco, USA) was used to investigate the functional blood vessels. Then, obtained tumor tissues were immediately stored in -80 °C.

### In vivo antitumor study

Mice were administered PBS, AE-MGNPs (iv), α-CTLA-4 (ip), or a combination of AE-MGNPs (iv) and α-CTLA-4 (ip) (Melittin 5 mg/kg, GSNO 0.5 mg/kg, α-CTLA-4 100 µg/mice) according to the scheme explained below. The longest diameter (A) and shortest diameter (B) of tumors in different groups were read with a caliper to determine the tumor volumes (calculated as: π/6 × A × B^2^) were recorded every 2 days. Tumors were cut to make sections for hematoxylin and eosin (HE) staining, the TUNEL assay, and IHC analysis. A in formula represented the longest diameter and B in formula represented the shortest diameter.

### Safety evaluation

Moreover, blood routine examination and blood biochemistry levels were detected. Histological sections staining was used to evaluate the damage in major organs. All the mouse experiments had achieved the approval from the Ethics Committee of the CAS Key Laboratory for Nano-Bio Interface, Suzhou Institute of Nano-Tech and Nano-Bionics, and Chinese Academy of Sciences.

### In vivo biodistribution studies

4 × 10^6^ cells (CT26 cells/CAFs: 3/1) were confused and inoculated into the right side of BALB/c mice. Then, DiR-NPs or AE-DiR-NPs (at a dose of DiR:0.4 mg/kg) were injected intravenously to assess the living-body fluorescence images via in vivo imaging system (IVIS Spectrum, Massachusetts, USA) at a fixed time point. At the end of the experiment, tumors as well as the major organs were obtained for ex vivo imaging for investigation of tissue distribution.

### Statistical analysis

All experiments were performed at least three times. Prism 8.0 and SPSS19.0 were used for statistical significance analysis, which was statistically significant if P value smaller than 0.05.

## Results and discussion

### The preparation and characterization of AE-MGNPs

**Fig. 1:**
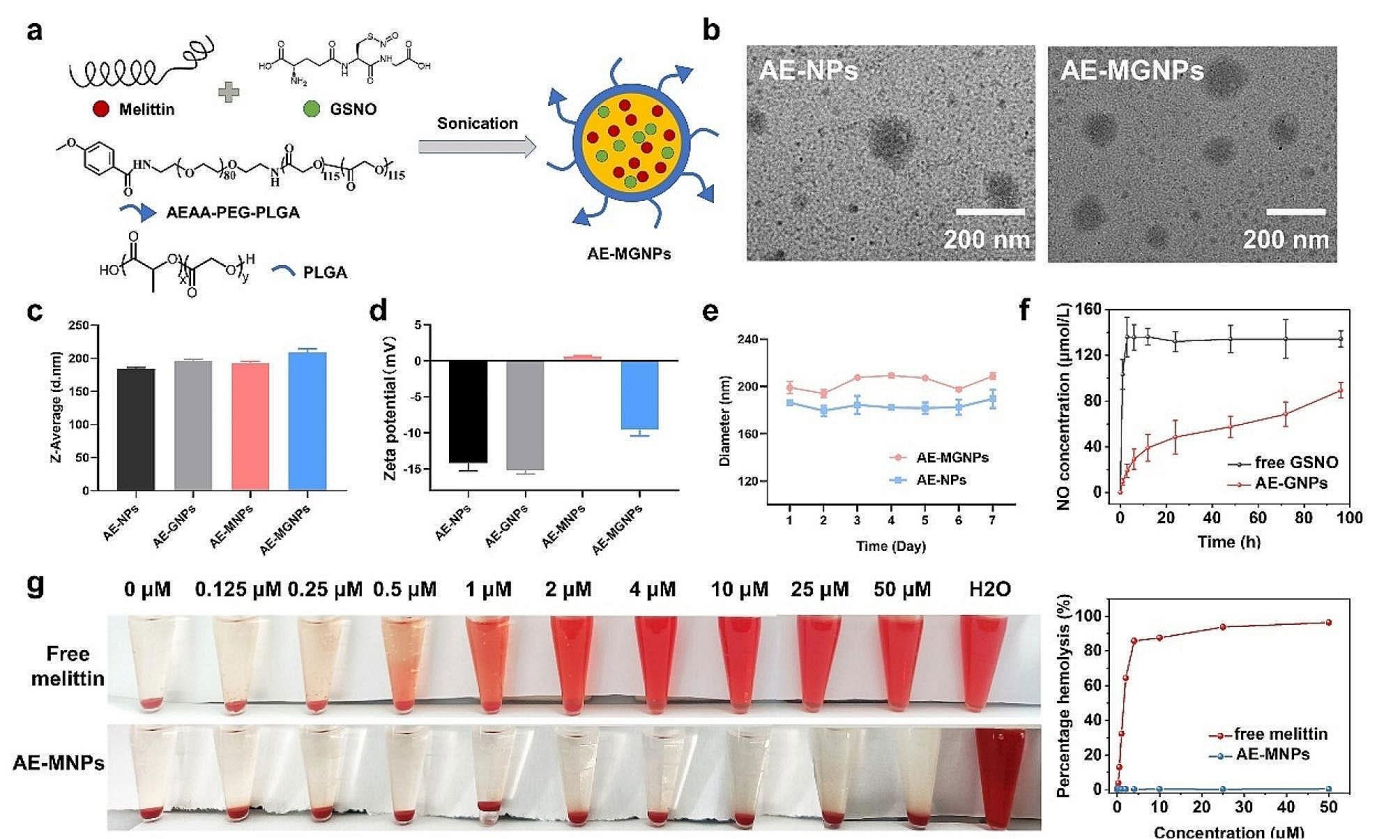
Synthesis and characterization of AE-MGNPs. (**a**) Efficient co-encapsulation of melittin and GSNO into PLGA NP using a solvent volatilization technique. (**b**) TEM morphology of AE-NPs and AE-GMNPs. (**c**) Z-Average and (**d**) ζ potential of AE-NPs, AE-MNPs, AE-GNPs and AE-MGNPs. (**e**) Time-dependent size stability of AE-MGNPs and AEAA-NPs in PBS at 4 °C. (**f**) NO release profiles of free GSNO and AE-GNPs. (**g**) Hemolysis assays for free Melittin, and AE-MNPs in RBC. Data are shown as the means ± SD (*n* = 4)

The melittin/GSNO-loaded nanoemulsions (AE-MGNPs) were gained through solvent evaporation method by mixing poly (lactic-co-glycolic acid)-poly (ethylene glycol)-aminoethyl anisamide (PLGA-PEG-AEAA) and poly (lactic-co-glycolic acid) (PLGA) with the addition of melittin and GSNO (Fig. 1a). Melittin-loaded nanoemulsions (AE-MNPs), GSNO-loaded nanoemulsions (AE-GNPs) and nanoemulsions without the inclusion of any drugs (AE-NPs) were also prepared for contrast. Transmission electron microscope (TEM) imaging clearly revealed the spherical structures of AE-NPs and AE-MGNPs (Fig. 1b), and dynamic light scattering (DLS) assay indicated that AE-MGNPs had a slightly larger hydrodynamic size in comparison to AE-NPs, AE-MNPs and AE-GNPs (Fig. 1c). The average sizes of AE-NPs and AE-MGNPs suggested no significant difference after 7 days in PBS, illustrating the outstanding long-term stability of these nanoemulsions (Fig. 1e). In addition of structural description, we investigated the drug-loading efficiency of these nanoemulsions. The EE% of melittin and GSNO in AE-MGNPs were calculated to be 41.92 ± 2.21% and 46.03 ± 2.03%, respectively, while the DL% of melittin and GSNO in AE-MGNPs were 20.1 ± 0.73% and 2.25 ± 0.87%, respectively (Table 1). Additionally, the potential of the resulting nanoemulsions for sustained drug release was explored. The release of nitrite (NO2^−^) exhibited a sustained pattern from AE-GNPs pattern, in comparison to the burst release observed with free GSNO (Fig. 1f). This sustained release characteristic is well-suited for continuous and long-term administration of NO in line with the blood circulation profile. Another important property of the nanoemulsions was that the highly positive charges of free melittin was shielded to form nanoemulsions with an approximately neutral zeta potential (0.631 ± 0.109 mV, *n* = 3) (Fig. 1d), strongly suggesting that systemic administration of melittin as nanoemulsions reduced its toxicity. To validate the hypothesis, the hemolytic behaviour of free melittin and AE-MNPs was detected at various concentration of melittin, up to 50 µM, with red blood cells (RBC). As shown in Fig. 1g, free melittin induced the complete lysis of RBC even at a low concentration (2 µM), whereas negligible hemolysis of RBCs occurred in the samples of AE-MNPs at a series of concentrations ranging from 0.125 to 50 µM (0.45 ± 0.3%, *n* = 3), confirming the lower toxicity of systemic administration of these nanoemulsions.

### The determination of CAFs targeting ability of AEAA ligand

**Fig. 2:**
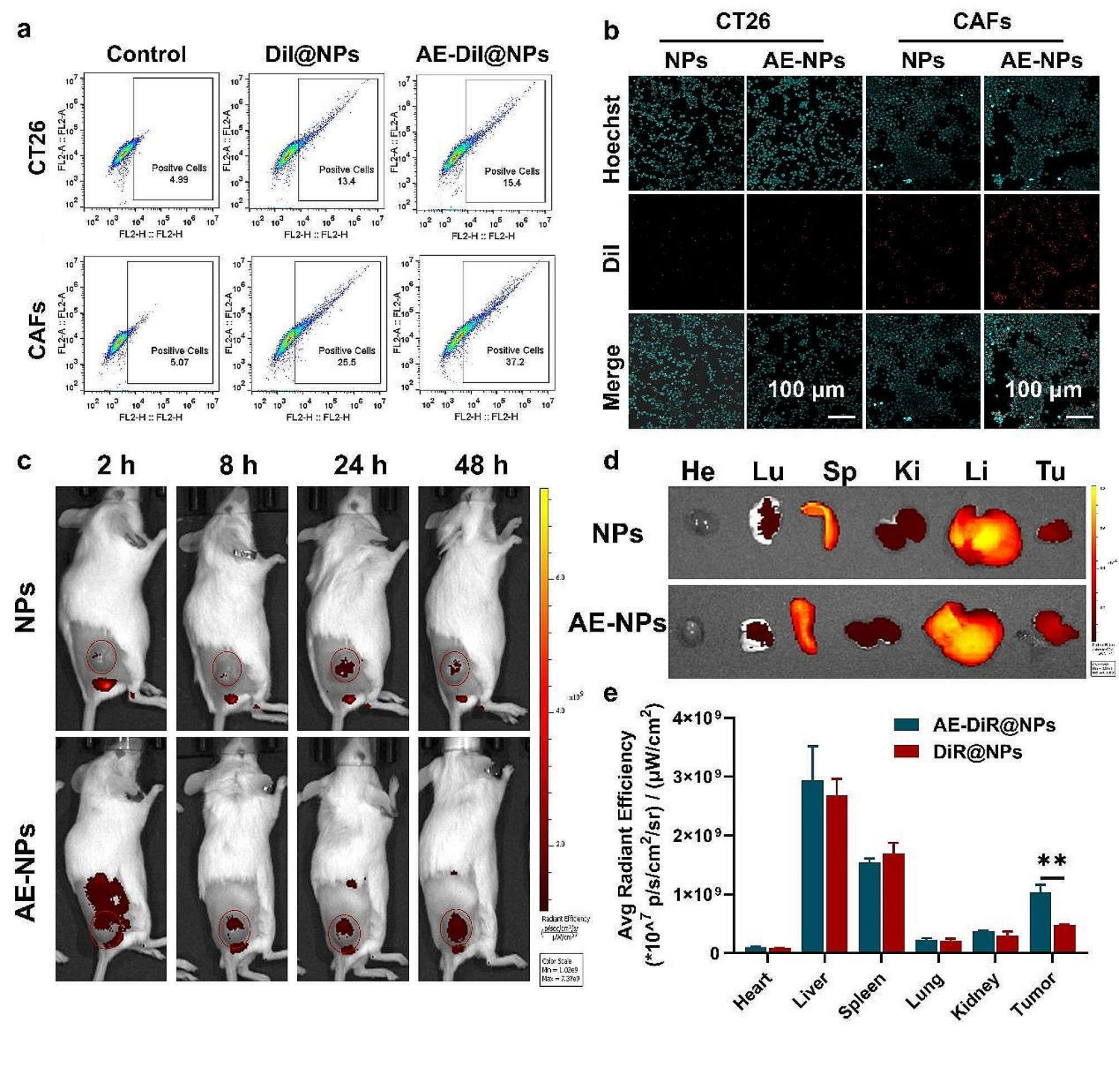
Biodistribution of NPs and AE-NPs in vitro and in vivo. (**a**) Flow binding diagram of CT26 and CAFs treated with DiI@NPs or AE-DiI@NPs for 1 h, respectively. Normal cells were served as negative controls. (**b**) Representative fluorescence microscopy images of CT26 and CAFs after treated using DiI-labeled NPs or AE-NPs for 1 h. (**c**) Living body images of CT26 tumor-bearing mice were acquired at fixed timepoint. The tumor area is marked in red circles. (**d**) Representative fluorescence images of excised tumors and major organs ex vivo. Heart (He), lung (Lu), spleen (Sp), kidney (Ki), liver (Li) and tumor (Tu). (**e**) The statistics of corresponding average radiation efficiency in five important organs and excised tumors. Scale bar, 100 μm. *P < 0.05, **P < 0.01

Melittin has been widely reported preclinically as an anticancer drug owing to its lytic activity on many types of cancer cells. However, its nonspecific attack on lipid membranes restricts its clinical applicability. The limitation can be surmounted by delivering melittin into the tumor microenvironment (TME) using a targeting ligand, AEAA, to target the sigma-1 receptor which is overexpressed on CAFs and many highly transferable tumor cells [28]. Therefore, the CAF-targeting ability of AE-NPs was investigated in vitro. CAFs were obtained via triggering the differentiation of NIH/3T3 using TGF/β1 (10 ng/mL, 48 h). The high expression of CAF biomarkers, including α-SMA and FAP-α suggested the successful generation of CAFs (Fig S1-2). Subsequently, flow cytometry and CLSM assay were conducted to investigate the cellular uptake of DiI-labeled nanoemulsions with or without AEAA modification in CT26 cells and CAFs, respectively. As shown in Fig. 2a-b and S3-4, both CAFs and CT26 cells efficiently took up AE-NPs and NPs after 1 h of co-incubation. AE-NPs revealed a higher cellular internalization efficiency compared with NPs in CAFs, whereas the cellular uptake of AE-NPs was similar to NPs in CT26 cells. Additionally, more AE-NPs were phagocytosed by CAFs compared to CT26 cells, which was potentially due to higher sigma-1 receptor expressed on CAFs, as confirmed by qPCR and CLSM (Fig S5-6). Encouraged by the remarkably targeting outcomes in vitro, the biodistribution of DiR-labeled NPs and AE-NPs was detected in both major organs and resected tumors via IVIS Spectrum. Both AE-DiR@NPs and DiR@NPs gathered prominently in the reticuloendothelial system and tumor tissues. Furthermore, the in vivo targeting facilitated by the AEAA ligand led to a greater accumulation of AE-DiR@NPs in tumor tissues in comparison to DiR@NPs (Fig. 2c-e). These results indicate that AEAA ligand improves the targeting capabilities of the nanoemulsions in CAFs.

### AE-MGNPs exhibit selective cytotoxicity toward cancer cells

**Fig. 3:**
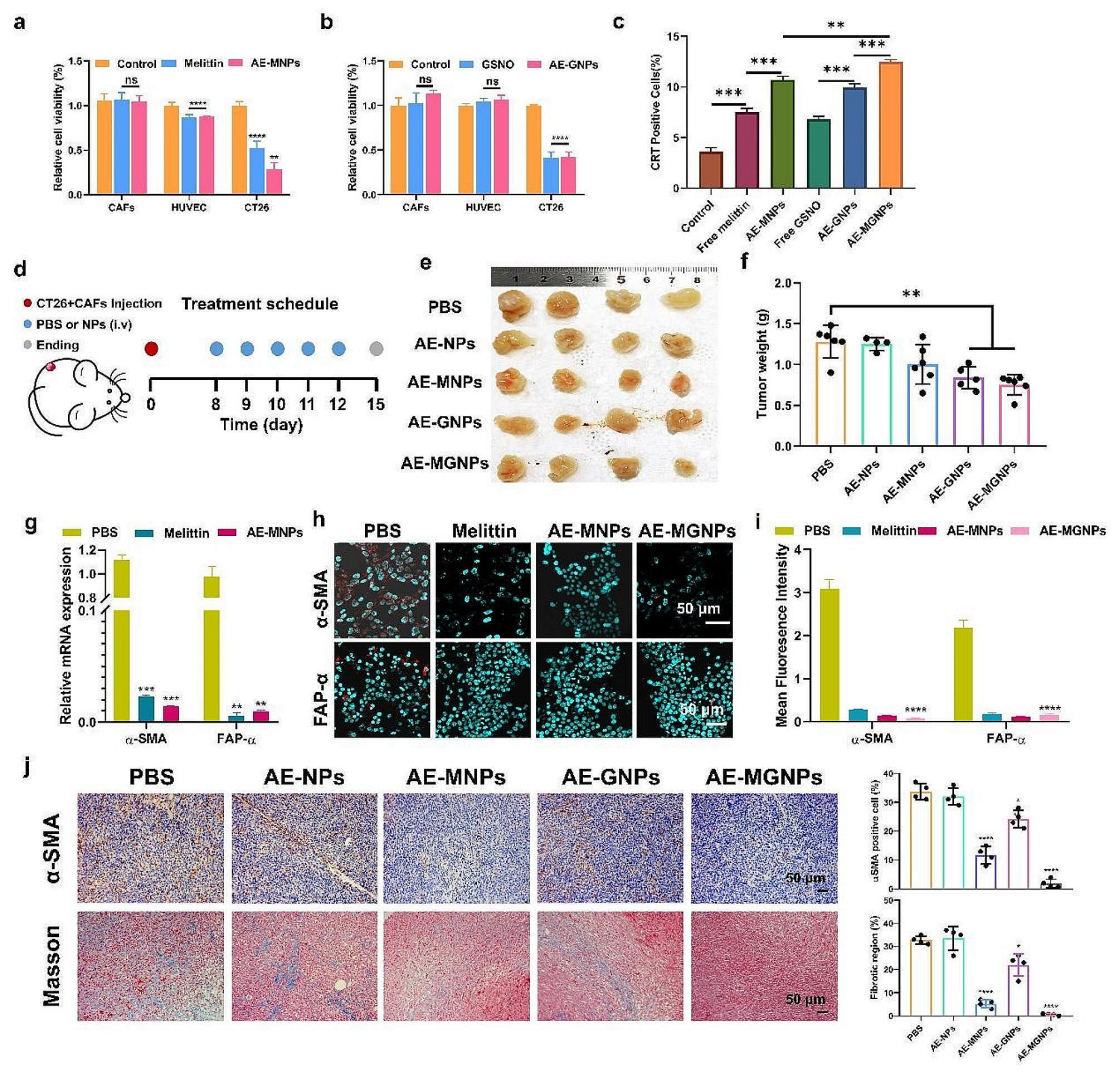
AE-MGNPs inhibited the tumor growth and remodeled the tumor microenvironment. Cell viability assays assessing the cytotoxicity of (**a**) AE-MNPs and (**b**) AE-GNPs in CAFs, CT26 and HUVEC after 72 h treatment at a concentration of melittin (4 µg/mL) and GSNO (29.99 µg/mL). Free melittin and GSNO were used as negative control. (**c**) The percentage of CRT-positive cells after 24 h of exposure. (**d**) Scheme of anti-cancer and TME remodeling evaluation in CT26 tumor-bearing mice. (**e**) Photographs and (**f**) tumor weight of excised tumors on day 15. (**g**) Melittin delivery reverses the activity of CAFs at the cellular level and inhibits the mRNA expression of α-SMA and FAP-α in CAFs. Untreated CAFs cells were used as controls. (**h**-**i**) Fluorescence microscopy images suggested the expression of α-SMA and FAP-α in CAFs after incubation using free melittin, MNPs or MGNPs. Untreated CAFs cells were served as negative controls. Scale bar, 50 μm. (**j**) Immunohistochemical staining of CAFs with α-SMA (upper) and collagen with Masson Trichrome (down) in tumor sections. Scale bar, 50 μm. *P < 0.05, **P < 0.01, ***P < 0.005, ****P < 0.001, ns No significance

The AE-NPs induced a negligible cytotoxicity towards the three types of cell lines (CT26, CAFs and HUVEC) even at a high dose of 500 µg/mL, forecasting the excellent biocompatibility of the prepared nanoemulsions (Fig S7). Considering the complexity of TME structure and function, Melittin and GSNO were expected to decrease the viability of cancer cells while reprograming TME without killing CAFs or other TME cells including vascular endothelial cells. Cancer cells have been found to be more sensitive to melittin than CAFs and HUVEC [29]. In consequence, the optimal concentrations of melittin and GSNO were determined using CT26, CAFs and HUVEC. As shown in Fig S8a-b, free melittin and GSNO showed a concentration-dependent cytotoxicity towards the three types of cells. Furthermore, the cytotoxic effect of both melittin and GSNO on CT26 cells was much stronger than that in CAFs and HUVEC. The IC50 values (melittin: 4 µg/mL; GSNO: 30 µg/mL) observed with CT26 cells was safe for CAFs and HUVEC cells. Therefore, 4 µg/mL melittin and 30 µg/mL GSNO were chosen as the optimum dose to prepare nanoemulsions for subsequent cells treatments. As expected, the prepared AE-MNPs and AE-GNPs showed a significantly reduced cytotoxicity towards CAFs and HUVEC similar to free melittin and GSNO after 72 h of co-incubation. However, approximately half of the CT26 cells were killed by this treatment (Fig. 3a-b). Additionally, AE-MNPs exhibited higher cytotoxicity in CT26 cells compared to free melittin, which was possibly due to the easier cellular internalization of nanoemulsions and greater sensitivity of CT26 cells to melittin. These results indicated that AE-MNPs and AE-GNPs could selectively kill cancer cells and did not affect CAFs and HUVEC. As classic indicators of the immunogenic cell death (ICD), calreticulin (CRT) exposure on the cell surface was gauged in vitro to investigate the ability of AE-MGNPs to induce immunogenic phenotypes [22]. As shown in Fig. 3c, AE-MNPs induced a greater proportion of CRT-positive cells compared to free melittin, and a similar trend was observed for free GSNO and AE-GNPs. Furthermore, the highest number of CRT-positive cells was observed in the AE-MGNPs treatment groups. These results indicated that AE-MGNPs induced the highest percentage of CRT-positive cells, suggesting that AE-MGNPs could be used as an anti-tumor vaccine to promote the maturation of antigen-presenting cells and activation of cytotoxic T lymphocytes (CTLs).

### AE-MGNPs inhibit tumor progression

The antitumor effects of AE-MGNPs were further investigated in CAFs-rich CT26 tumor-bearing mice (Fig. 3d). As shown in Fig. 3e-f, AE-MGNPs induced the greatest inhibition on the tumor volume and weight compared to other groups (*p* < 0.01). Furthermore, there were no significant differences in body weight (Fig. S9), hematological factors, serum biochemical factors (Fig. S10) and organ histology (Fig. S11) of the liver, spleen, kidneys, heart, and lungs in the mice after treated with AE-MGNPs, suggesting that the repeated intravenous injections of AE-MGNPs did not cause obvious toxicity or side effects in vivo. The antitumor effect of AE-MGNPs can be attributed, at least in part to the reverse of the activation state of CAFs, given that activated CAFs are known to exert tumor-promoting and immunosuppression effects [8, 10]. Therefore, the ability of AE-MGNPs to reprogram CAFs was investigated in vitro and in vivo. As shown in Fig. 3g-i, the expression levels of α-SMA and FAP-α were significantly decreased in CAFs after incubated with free melittin, AE-MNPs and AE-MGNPs. Consistent results were observed in vivo. AE-MNPs and AE-MGNPs induced a significant reduction of α-SMA positive CAFs in the TME (Fig. 3j, up). Additionally, collagen secreted from activated CAFs, was also remarkably reduced in the AE-MNPs and AE-MGNPs treatment groups (Fig. 3j, down). In contrast, AE-NPs and AE-GNPs had no significant effect on the α-SMA expression level of CAFs and the production of collagen. These results indicated that melittin-loaded nanoemulsions effectively inhibited the activation state of CAFs. Melittin was reported to reduce the fibrotic properties in both liver and renal via inhibiting TGF-β-induced pro-fibrotic gene expression, which may give interpretation to the reprogramming function of melittin in CAFs cells [19, 30]. Interestingly, AE-MGNPs exhibited a better inhibition on the activation state of CAFs than AE-MNPs in vivo, which could potentially be attributed to the enhanced drug delivery induced by NO.

### AE-MGNPs normalize the tumor blood vessels

**Fig. 4:**
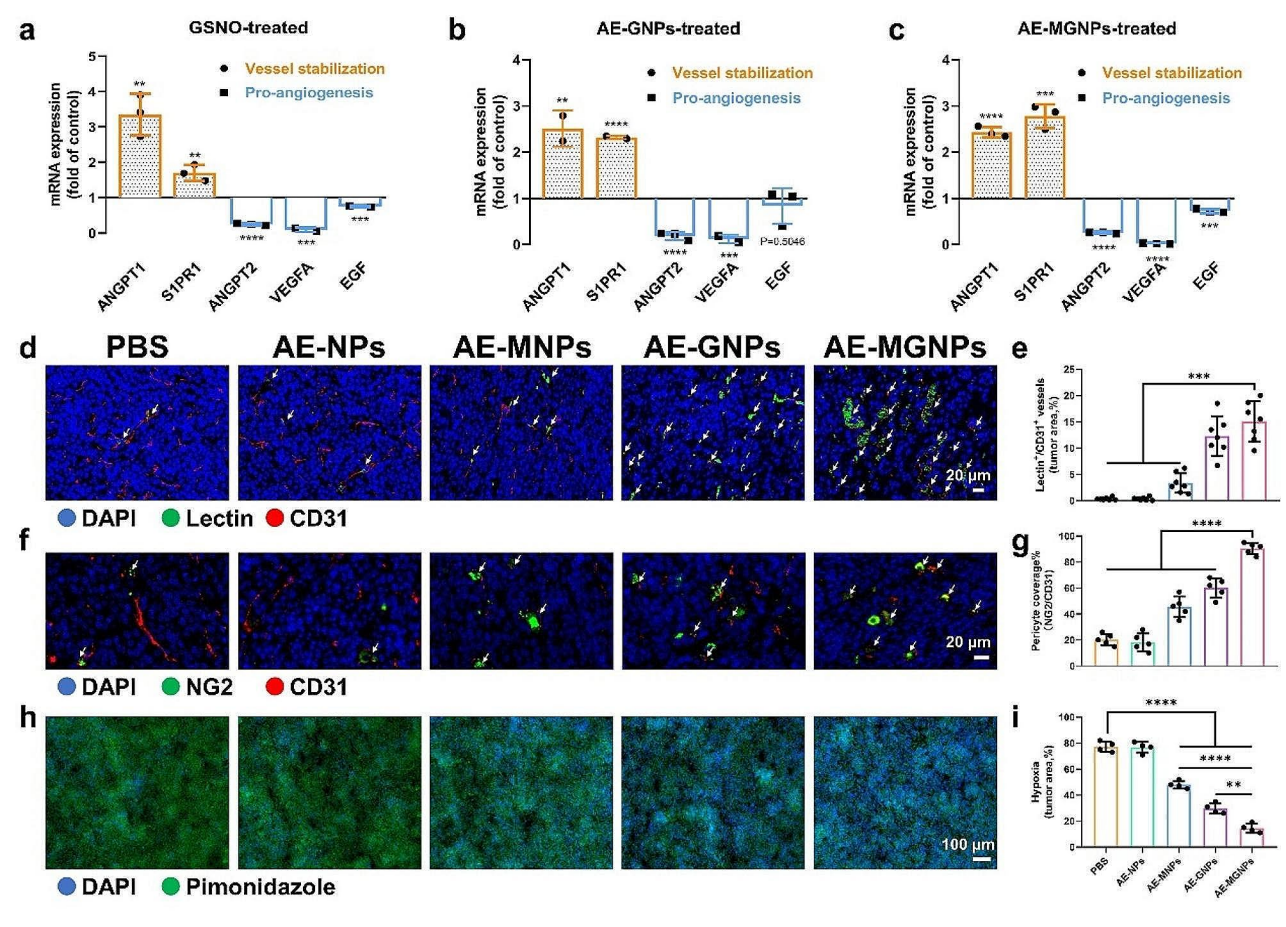
AE-MGNPs normalizes tumor vasculature in CRC. (**a**-**c**) Relative expression of vessel-stabilizing and pro-angiogenic factors in HUVECs after incubating with free GSNO, AE-GNPs, and AE-MGNPs measured by qPCR. Results were exhibited as fold change compared to the untreated cells. (**d**-**e**) Tumor vessel perfusion in CRC tumors after five consequently treatments with AE-MNPs, AE-GNPs or AE-MGNPs. Vessel perfusion in tumors were marked using white arrow. Scale bars, 20 μm. (**f**-**g**) Quantification of pericyte coverage (fraction of area covered) in CRC after administration with AE-MNPs, AE-GNPs or AE-MGNPs. CD31^+^ endothelial cells were stained red and NG2^+^ pericytes were stained green. Scale bars, 20 μm. (**h**-**i**) Proportion of pimonidazole^+^ areas were used as a marker for hypoxia evaluation in CRC after treatment with AE-MNPs, AE-GNPs or AE-MGNPs. Scale bars, 100 μm. All above animals’ experiments were conducted at a dose of melittin 4 µg/mL and GSNO 30 µg/mL. **P < 0.01, ***P < 0.005, ****P < 0.001

NO plays a critical role in regulating vascular stabilization and maintaining the vascular function, which can improve drug permeation and alleviate tumor hypoxia [23, 31, 32]. To investigate the ability of AE-MGNPs to normalize the tumor blood vessels, the expression levels of five vascular stabilization-related genes were detected in HUVECs to evaluate the vascular normalization and vascular maturation [22]. As shown in Fig. 4a-c, three proangiogenic genes (*EGF*, *ANGPT2* and *VEGFA*) were downregulated whereas two vessel maturation-related genes (*ANGPT1* and *S1PR1*) were upregulated in the HUVECs after incubating with the free GSNO, AE-GNPs and AE-MGNPs. These results indicate that GSNO-loaded nanoemulsions induce a transformation of endothelial cells from a proangiogenic phenotype to a vascular-stabilizing feature. Besides the immediate impression of NO on vessel stabilization and angiogenesis, NO in perivascular space is known to normalize tipsy tumor blood vessel structure and function. Hence, the vessel normalization as well as mean vessel density (MVD) were observed in vivo after treatment with AE-MGNPs. As shown in Fig. 4, AE-MGNPs, AE-MNPs and AE-GNPs did not change tumor MVD, but significantly alleviated the vascular distortion, decreased structural heterogeneity as well as improved the functional perfusion (lectin^+^/CD31^+^ area) (Fig. 4d-e and S12) and vascular pericyte coverage (NG2^+^/CD31^+^ area) (Fig. 4f-g and S13) compared with the PBS and AE-NPs. Notably, AE-MGNPs showed a higher vessel normalization efficacy than AE-GNPs, which was likely because the inactivation of CAFs induced by melittin relieved the mechanical compression of ECM on blood vessels. Based on the CAFs reprogramming and vessel normalization, AE-MGNPs induced a prominent diminution in the hypoxic tumor area, evidenced by pimonidazole staining, indicating a diminution in malignant tumor progression and aggressiveness compared to the control groups (Fig. 4h-i), which might conduce to decreasing tumor malignant progression and aggressiveness.

### AE-MGNPs alleviate the immunosuppressive microenvironment and augment the antitumor immunotherapy

**Fig. 5:**
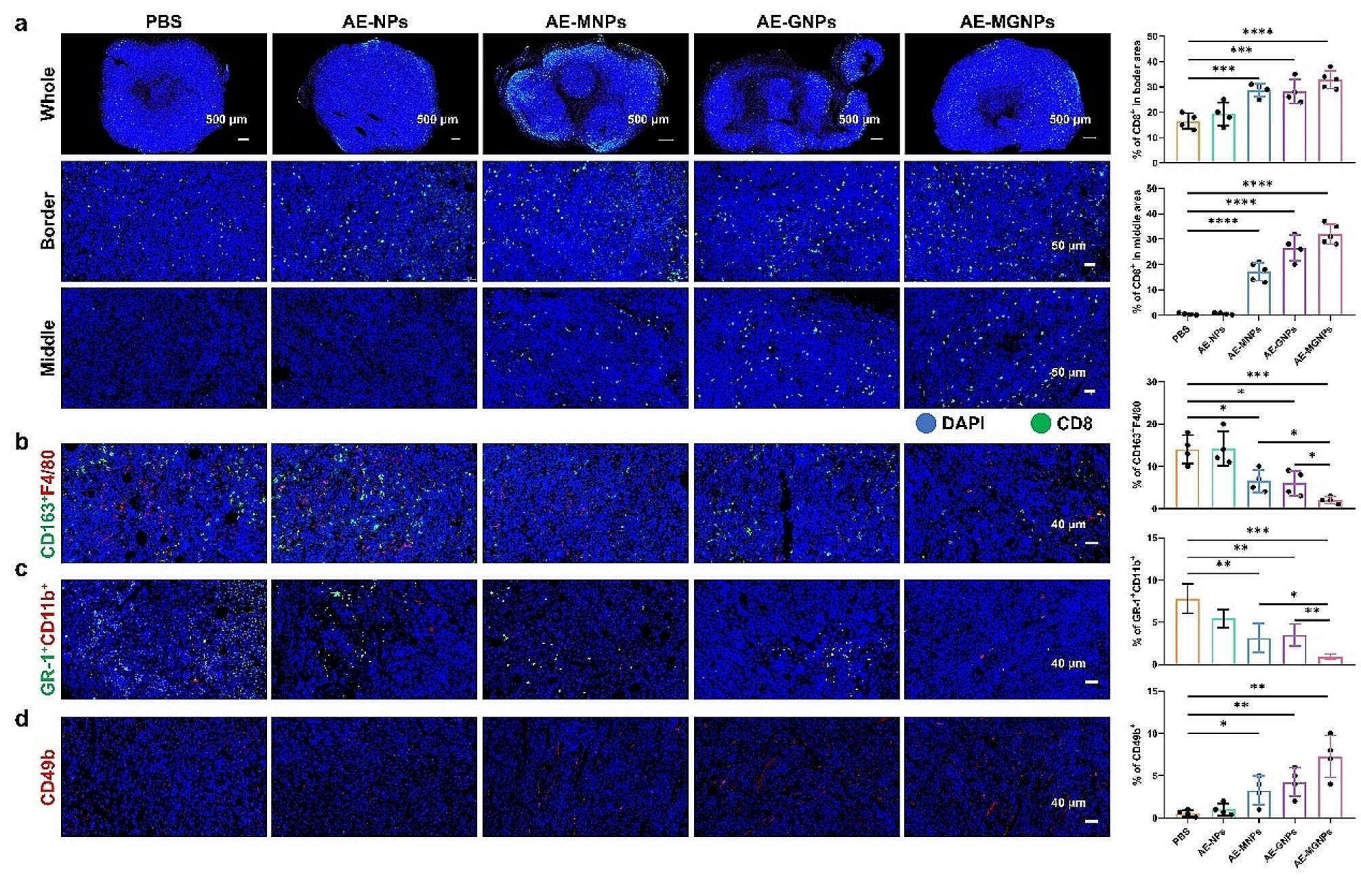
AE-MGNPs reverses the immunosuppressive microenvironment of colon cancer. (**a**) Total and regional tumor tissue sections obtained from mice that had been administrated with PBS, AE-NPs, AE-MNPs, AE-GNPs and AE-MGNPs were stained for CD8 and analyzed using CLSM. The proportions of (**b**) M-2 macrophages (**c**) MDSCs and (**d**) NK cells in tumor sections. *P < 0.05, **P < 0.01, ***P < 0.005

Aberrant tumor vasculatures and a highly dense ECM generate a protective barrier that impedes the tumor infiltration of CTLs [33, 34]. Therefore, the number and distribution of CD8^+^ T cells were detected in the tumor sections from CAFs-rich CT26 tumor-bearing mice after five consecutive injections of various formulations. AE-MNPs and AE-GNPs effectively improved the accumulation and penetration depth of CD8^+^ T cells in tumor tissues (Fig. 5a). The maximum number of CD8^+^ T cells infiltration was observed in tumors from AE-MGNPs-treated mice.

Functional CD8^+^ T cells play a pivotal role in the eradication of tumor cells, serving as the cornerstone of anti-tumor immunity. However, many cell types, including M2 macrophages, MDSC cells, CAFs and tumor cells, potentially inhibit CD8^+^ T cell-mediated tumor killing processes, including T cell activation, expansion, differentiation and infiltration [35]. Therefore, we investigated the tumoral infiltration of M2 macrophages and MDSC cells in tumor-bearing mice after treatment with AE-MGNPs. As shown in Fig. 5b-c, AE-MGNPs effectively decreased the content of both M2 macrophages and MDSCs compared with the control group, suggesting that AE-MGNPs virtually moderated the immunosuppressive TME by inhibiting the periodicity of immunosuppressive cells in tumor tissues. In addition, researchers have verified that NK cells played a dynamic role in anti-tumor immunotherapy [36]. Therefore, the infiltration of NK cells in treated tumors was increased in the AE-MGNPs treatment groups compared with that in the PBS and AE-NPs treatment groups (Fig. 5d), which suggested activation of the innate immune system. These results confirmed that AE-MGNPs ameliorated the immunosuppressive TME including improving the infiltration of CD8^+^ T cells and NK cells while inhibiting infiltration of immunosuppressive cells through reprogramming CAFs and normalizing blood vessels, which may potentially enhance the immunotherapeutic effect.

**Fig. 6:**
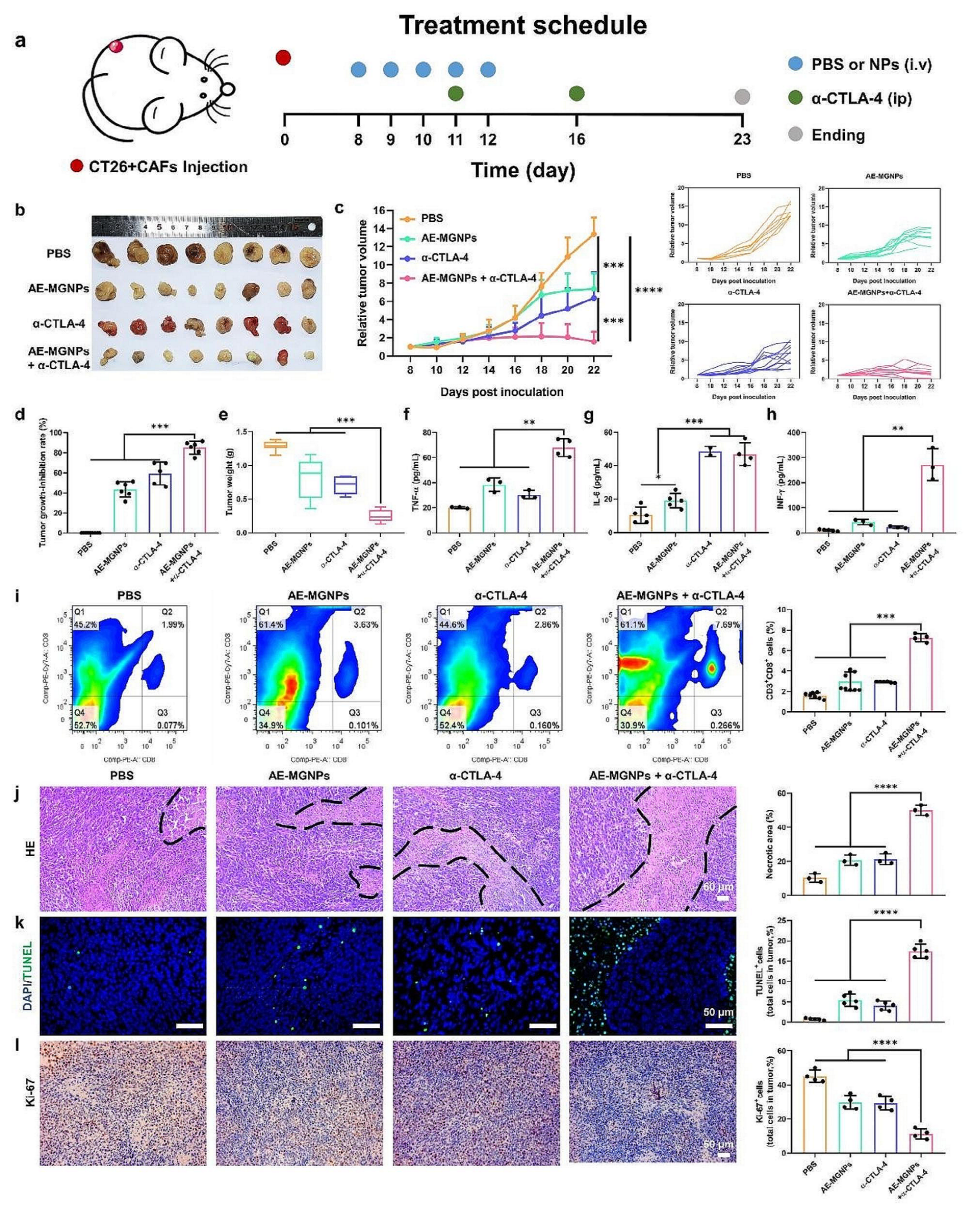
Combination of AE-MGNPs and α-CTLA-4 in CT26 tumor model. (**a**) AE-MGNPs and α-CTLA-4 combination treatment scheme. (**b**) Photographs of the excised tumors on day 23. (**c**) Tumor growth profiles after administration with PBS, AE-MGNPs, α-CTLA-4 or AE-MGNPs combined α-CTLA-4. (**d**) Tumor growth-inhibition rate and (**e**) tumor weight in the end of the experiments. After first α-CTLA-4 treatment (day 12), serum and primary tumor tissue were collected for the analysis of (**f**) TNF-α, (**g**) IL-6, (**h**) IFN-γ levels in serum and for analysis of the content of (**i**) CD3^+^/CD8^+^ T cells. (**j**) Representative images and statistical analysis of H&E staining (upper), TUNEL assays (middle), IHC analysis of the expression level of Ki-67 (down) in tumor sites. The area circled by the black line was the necrotic area of the tumor. Scale bar, 50 μm. **P < 0.01, ***P < 0.005, ****P < 0.001

To investigated whether AE-MGNPs sensitized tumors to immune checkpoint blockade therapy, we evaluated the antitumor effect of AE-MGNPs combined with α-CTLA-4 on CAF-rich CT26 tumor-bearing mouse models (Fig. 6a). As shown in Fig. 6b-e, compared to the moderate inhibition of tumor growth by the AE-MGNPs or α-CTLA-4 monotherapy, the combined treatment of AE-MGNPs and α-CTLA-4 dramatically slowed down the tumor progression as also evidenced by the smallest tumor volume of size of the combination therapy group among all treatment groups 23 days after the tumor inoculation. AE-MGNPs with α-CTLA-4 induced the most pronounced immune response including an increased ratio of CD8^+^ T cells (Fig. 6i) and pro-inflammatory cytokine production [37], such as tumor necrosis factor-α (TNF-α), interleukin-6 (IL-6), and interferon-γ (IFN-γ) (Fig. 6f-h). To explore whether AE-MGNPs induce the ICD effect, we detect the exposure of calreticulin (CRT) and the secretion of HMGB1 in tumors of mice after various treatments. As shown in Fig S14, AE-MGNPs plus α-CTLA-4 resulted in a highest level of CRT exposure and HMGB1 secretion, indicating that the combination of AE-MGNPs with α-CTLA-4 elicited a strong antitumor immune response. HE staining (Fig. 6j) and TUNEL assay (Fig. 6k) revealed that the combination therapy of AE-MGNPs and α-CTLA-4 induced higher necrosis and apoptosis compared to the AE-MGNPs or α-CTLA-4. The expression of Ki67 protein, a protein that serves as a cellular marker for proliferation [38], was markedly decreased in the tumor sections of mice treated with AE-MGNPs with α-CTLA-4 compared with those treated with AE-MGNPs or α-CTLA-4 group (Fig. 6l). These results confirmed that the combination of AE-MGNPs with immune checkpoint blockade elicited a robust anti-tumor immunity. Encouragingly, there was no obvious weight loss, serum biochemical change and histological abnormity occurred in all treatment groups (Fig S15-17), indicating the systemic toxicity induced by the combined treatments was not noticeable.

## Conclusion

In summary, CAF-targeted nanoemulsions, named AE-MGNPs, were developed for the co-delivery of melittin and GSNO. After optimizing the concentration of melittin and GSNO, AE-MGNPs selectively killed cancer cells without decreasing the viability of CAFs and vascular endothelial cells, and improved tumor immunogenicity by inducing ICD in cancer cells via CRT exposure. AE-MGNPs transformed the activated CAFs into a quiescent state, thus decreasing ECM deposition, relieving the compressed vessels, and further normalizing the tumor vessels through sustained NO release to improve T cell transportation. The synergistic modulation of the TME effectively mitigated the physical barriers imposed by excessive stroma and dysfunctional tumor vessels, thereby alleviating the immunosuppressive TME. Consequently, this modulation enhanced the infiltration of CD8 + T cells and NK cells, while reducing the frequency of immunosuppressive cells, including MDSCs and M2 macrophages. Additionally, AE-MGNPs combined with immune checkpoint inhibitors showed excellent tumor growth inhibition and reduced side effects in CAF-rich colorectal cancer models. Our study provides compelling evidence for the potential of a dual strategy involving the induction of ICD and modulation of the TME in enhancing the efficacy of checkpoint blockade immunotherapy. This finding holds significant promise for improving the response of patients to such therapeutic interventions.

### Electronic supplementary material

Below is the link to the electronic supplementary material.

Supplementary Material 1
