# Supplementary material for: Tumor microenvironment reprogramming combined with immunogenic enhancement by nanoemulsions potentiates immunotherapy
Source: J Nanobiotechnology. 2024 Apr 5;22:154. doi: 10.1186/s12951-024-02401-y (PMC10996274; doi:10.1186/s12951-024-02401-y)
Supplement: Supplementary file 1 — Supplementary Material 1 [file 12951_2024_2401_MOESM1_ESM.docx]

Fig.S1 The mRNA level of α-SMA and FAP-α in CAFs was detected by qPCR, GAPDH was used as internal control. **P<0.01, ***P<0.005.


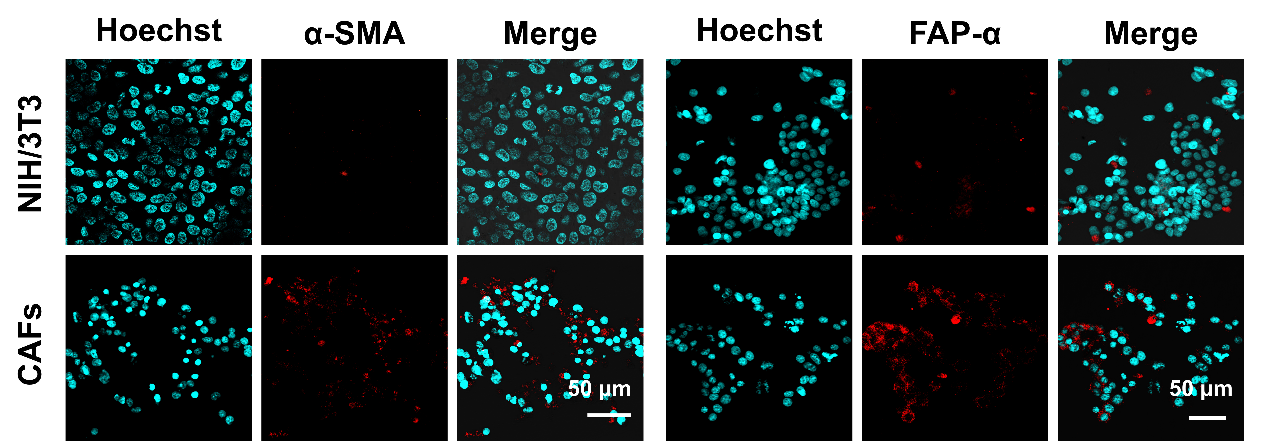


Fig.S2 The protein level of α-SMA and FAP-α in CAFs was detected by CLSM. Scale bar, 50 µm.


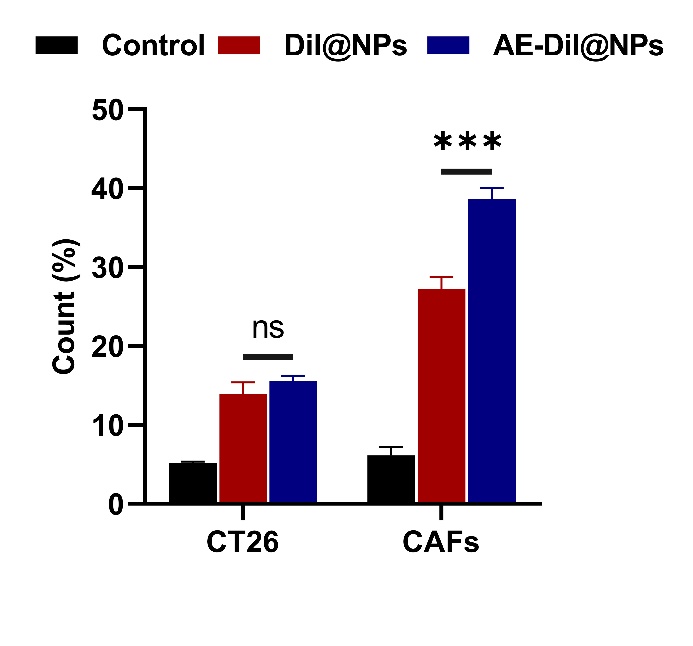


Fig.S3 Quantification analysis of average fluorescence intensity in CT26 and CAFs. ns No significance, ***P < 0.005.

Fig.S4 Mean fluorescence intensity of CT 26 cells and CAFs cells after incubation with DiI-labeled NPs or AE-NPs for 1 h. *P<0.05, ****P < 0.001.

Fig.S5 The mRNA level of Sigma-1R in CAFs was detected by qPCR, GAPDH was used as internal control. **P<0.01.

Fig.S6 The protein level of Sigma-1R in CAFs was detected by CLSM. Scale bar, 100 µm.

Fig.S7 Cytotoxicity of free AE-NPs against CT 26, CAFs and HUVEC cells after 72 h incubation at different concentrations.


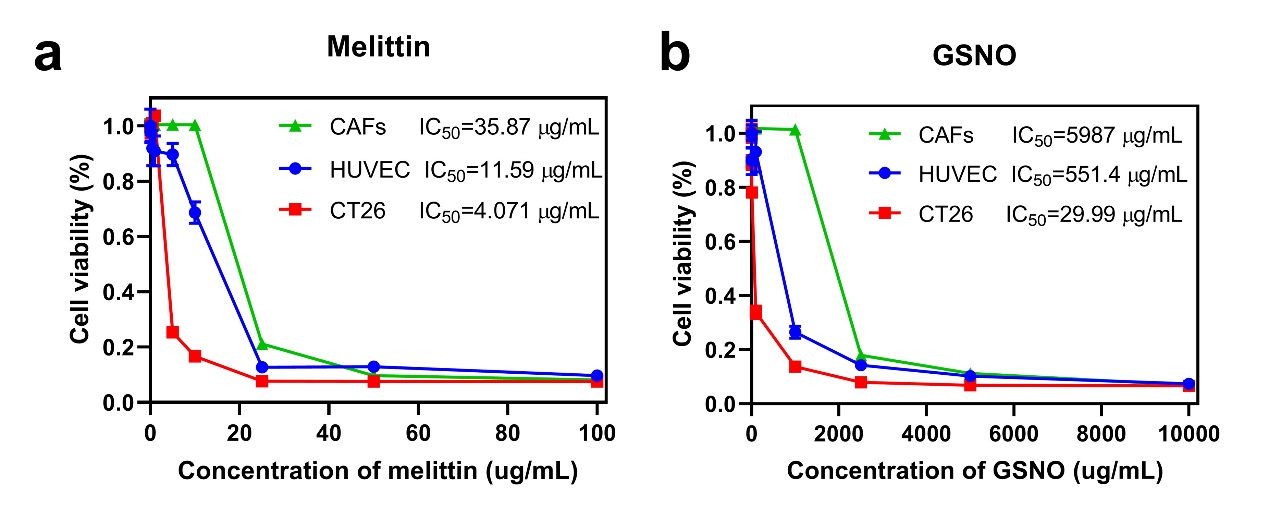


Fig.S8 IC50 of (a) free Melittin and (b) GSNO in CT26, CAFs and HUVEC cells.


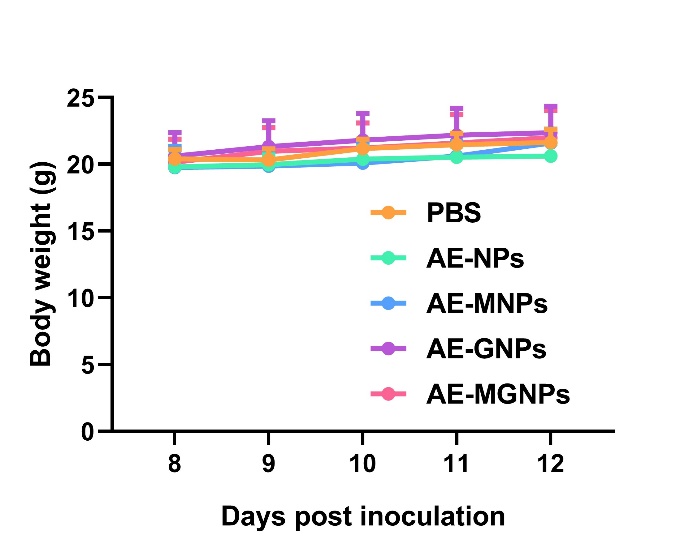


Fig.S9 Influences of various nanoemulsions on body weight of CT26 subcutaneous xenograft mice during TME remodeling.


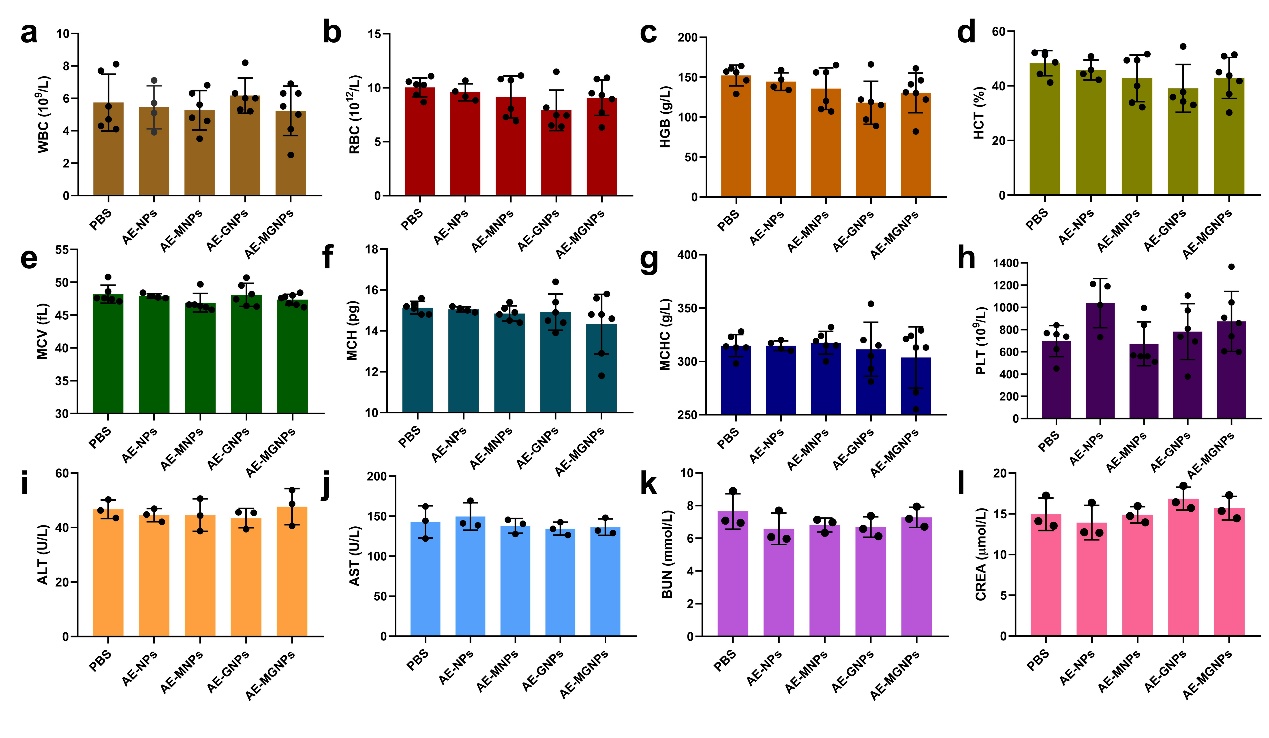


Fig.S10 Hematological tests and serum biochemicals tests of the mice after five days TME remodeling. (A) WBC white blood cells, (B) RBC red blood cells, (C) HGB hemoglobin, (D) HCT hematocrit, (E) MCV mean cell volume, (F) MCH mean corpuscular hemoglobin, (G) MCHC mean corpuscular hemoglobin concentration, (H) PLT blood platelet, (I) ALT alanine transferase, (J) AST aspartate transferase, (K) BUN blood urea nitrogen, (L) CREA creatinine. The data are presented as the mean ± S.D. (n=5)


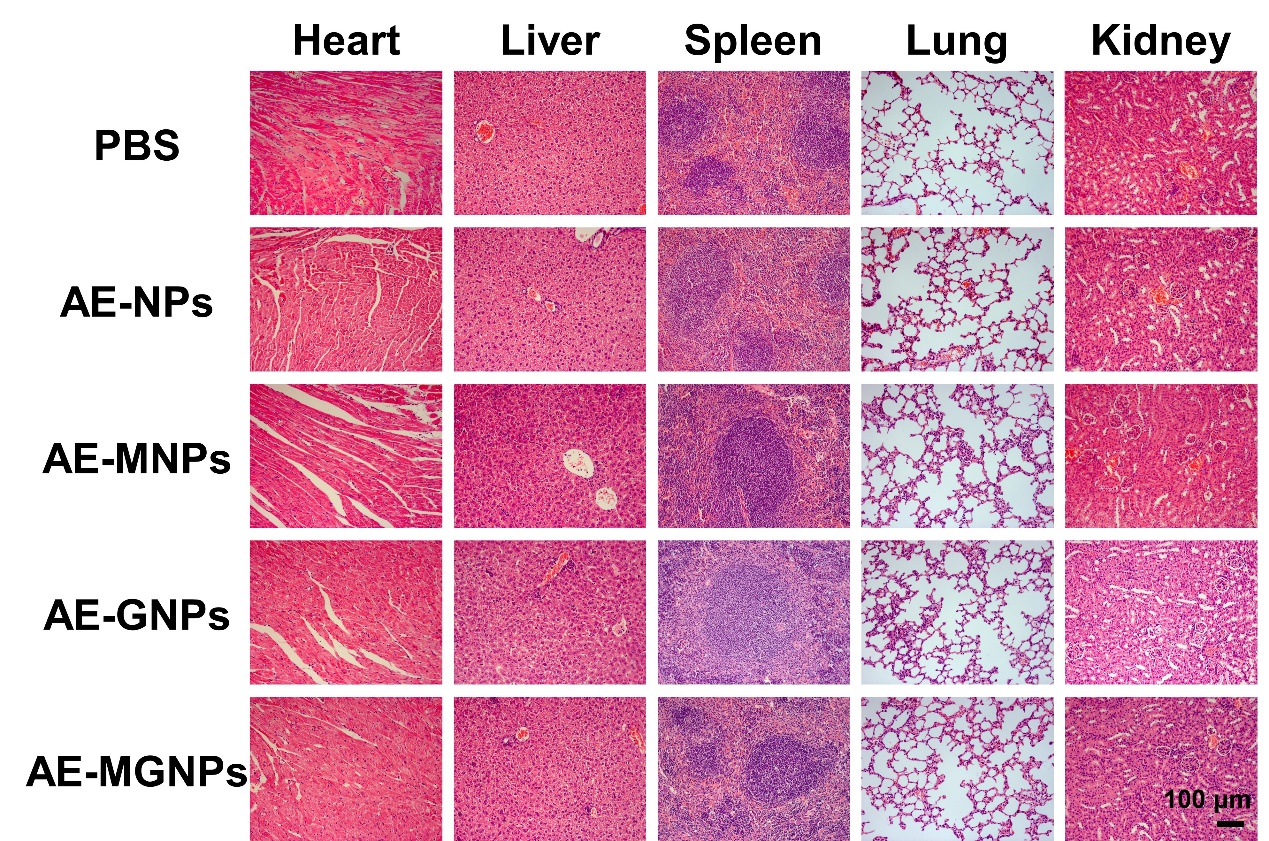


Fig.S11 Histological evaluation of the major organs, including the heart, liver, spleen, lungs and kidneys, of the CT 26 tumor-bearing mice after 5 days TME remodeling. Scale bar, 100 µm.


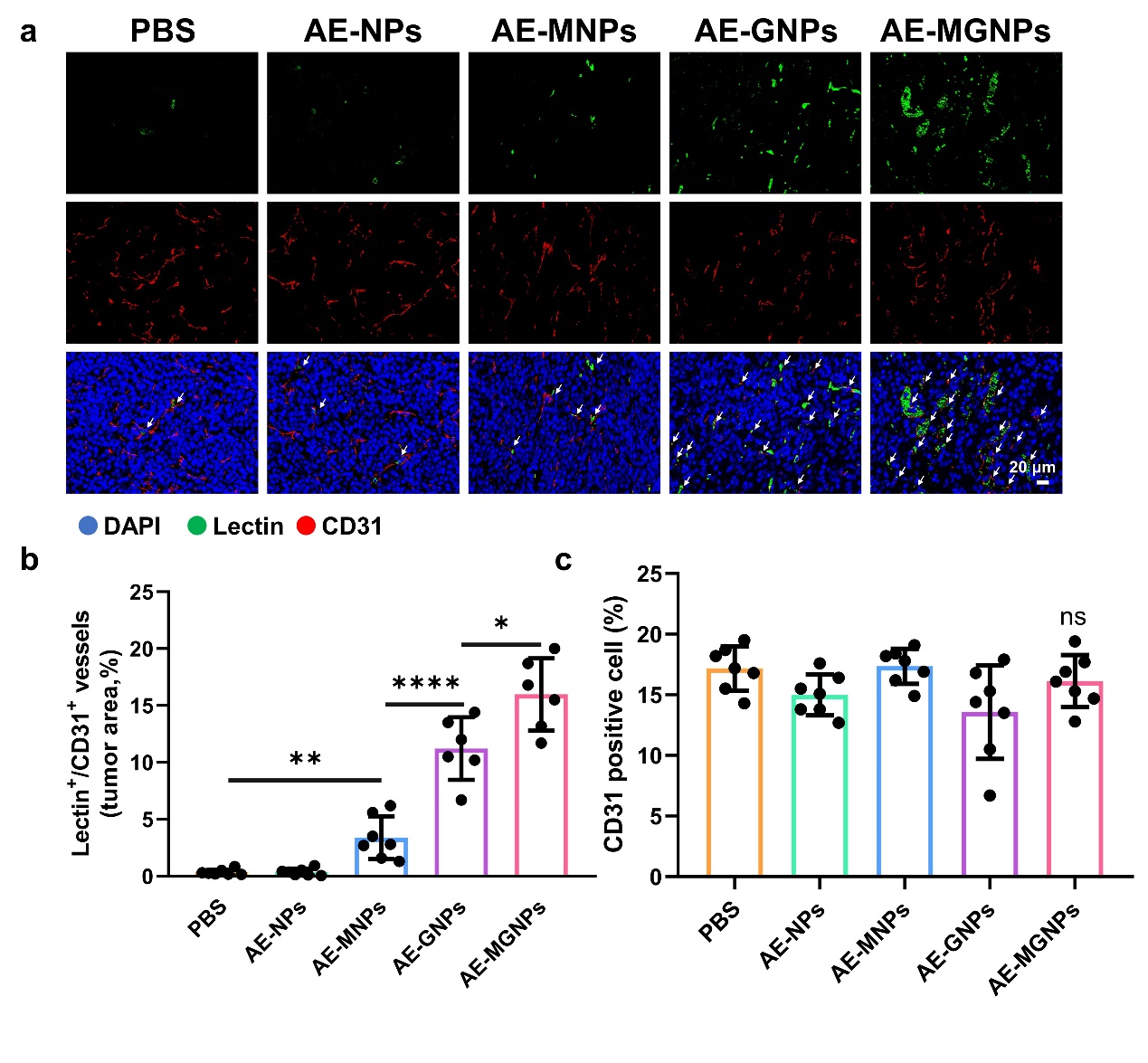


Fig.S12 Tumor vessel perfusion in CRC tumors after five consequently treatments with AE-MNPs, AE-GNPs or AE-MGNPs. (a) Vessel perfusion in tumors were marked with white arrowhead. (b) Quantification analysis of perfused functional tumor vessels in tumor sections. (c) Quantification analysis of MVD in tumor sections after treated with GSNO-loaded nanoemulsions. Scale bars, 20 μm. *P < 0.05, **P < 0.01, ****P < 0.001, ns No significance.


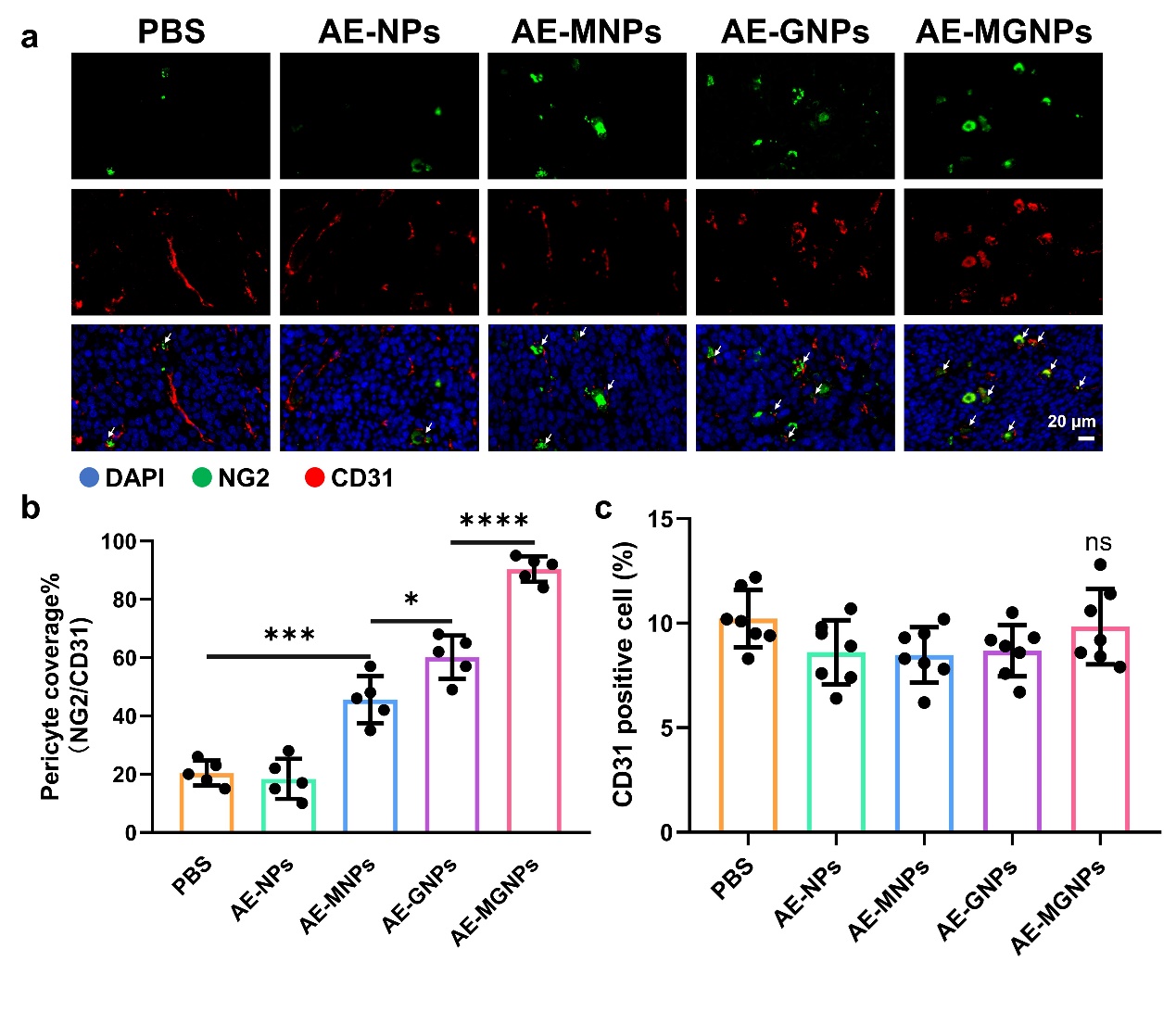


Fig.S13 Tumor vessel pericyte coverage in CRC tumors after five consequently treatments with AE-MNPs, AE-GNPs or AE-MGNPs. (a) Vessel pericyte coverage in tumors were marked with white arrowhead. (b) Quantification analysis of pericyte coverage tumor vessels in tumor sections. (c) Quantification analysis of MVD in tumor sections after treated with GSNO-loaded nanoemulsions. Scale bars, 20 μm. *P < 0.05, ***P < 0.005, ****P < 0.001, ns No significance.


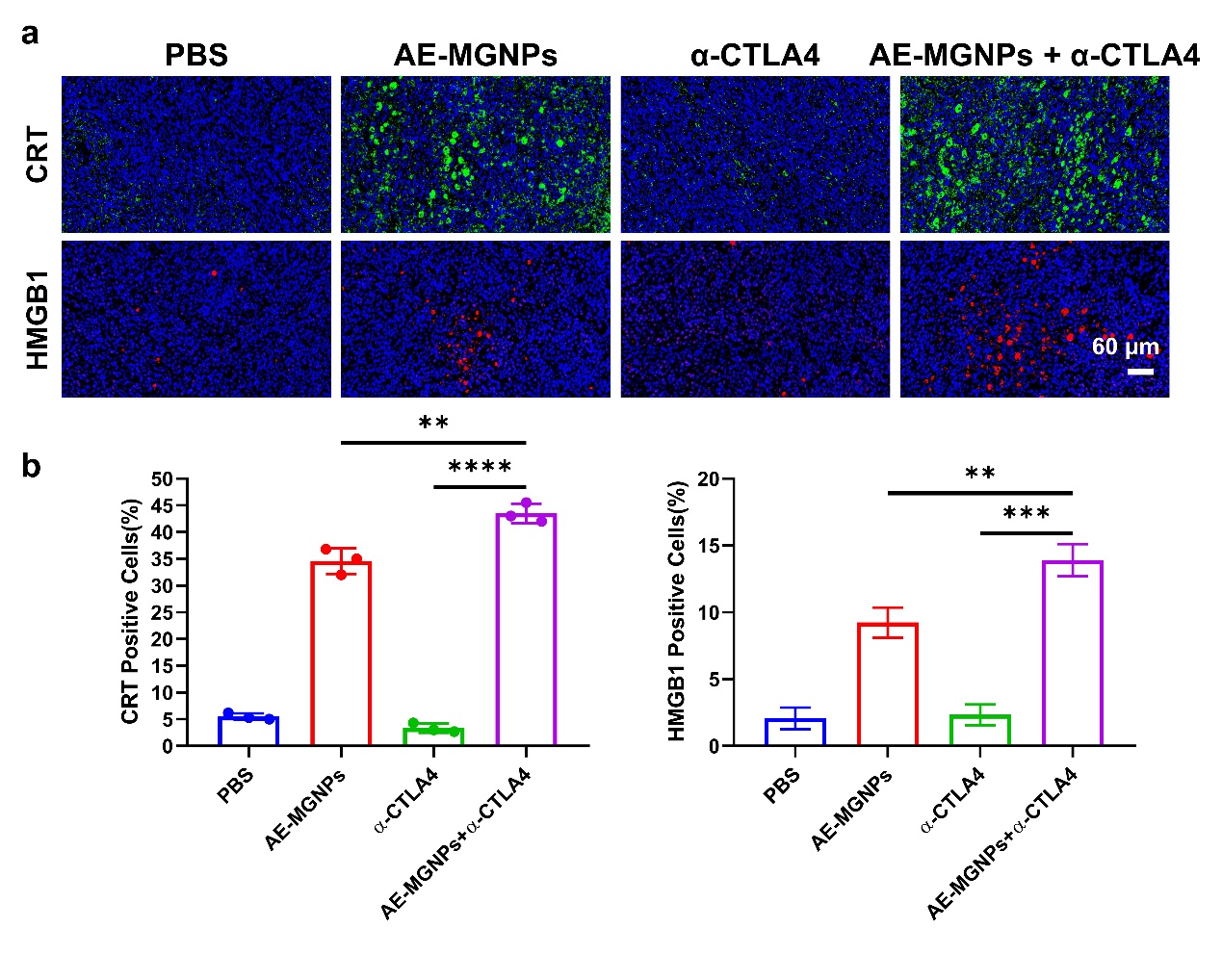


Fig.S14 (a) Representative images and (b) statistical analysis of CRT staining (upper) and HMGB1 assays (down) in tumor sites. Scale bar, 60 μm. ***P < 0.005, ****P < 0.001, ns No significance.


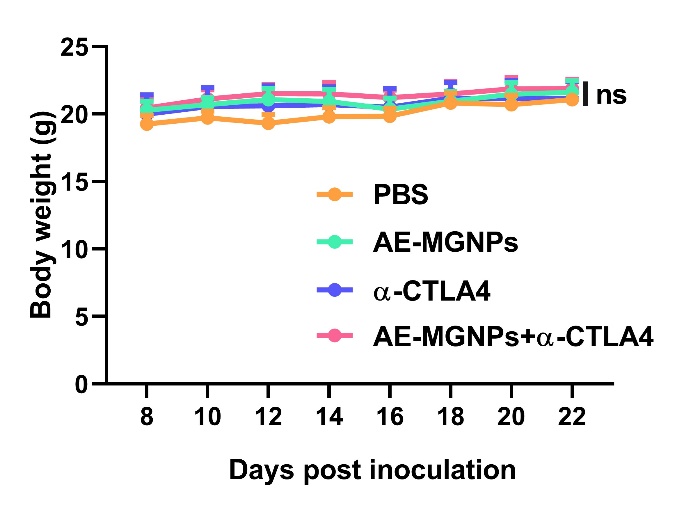


Fig.S15 The body weights of the CT 26 tumor-bearing mice from each group after 23 days of treatment. The data are presented as the mean ± S.D. (n = 5).


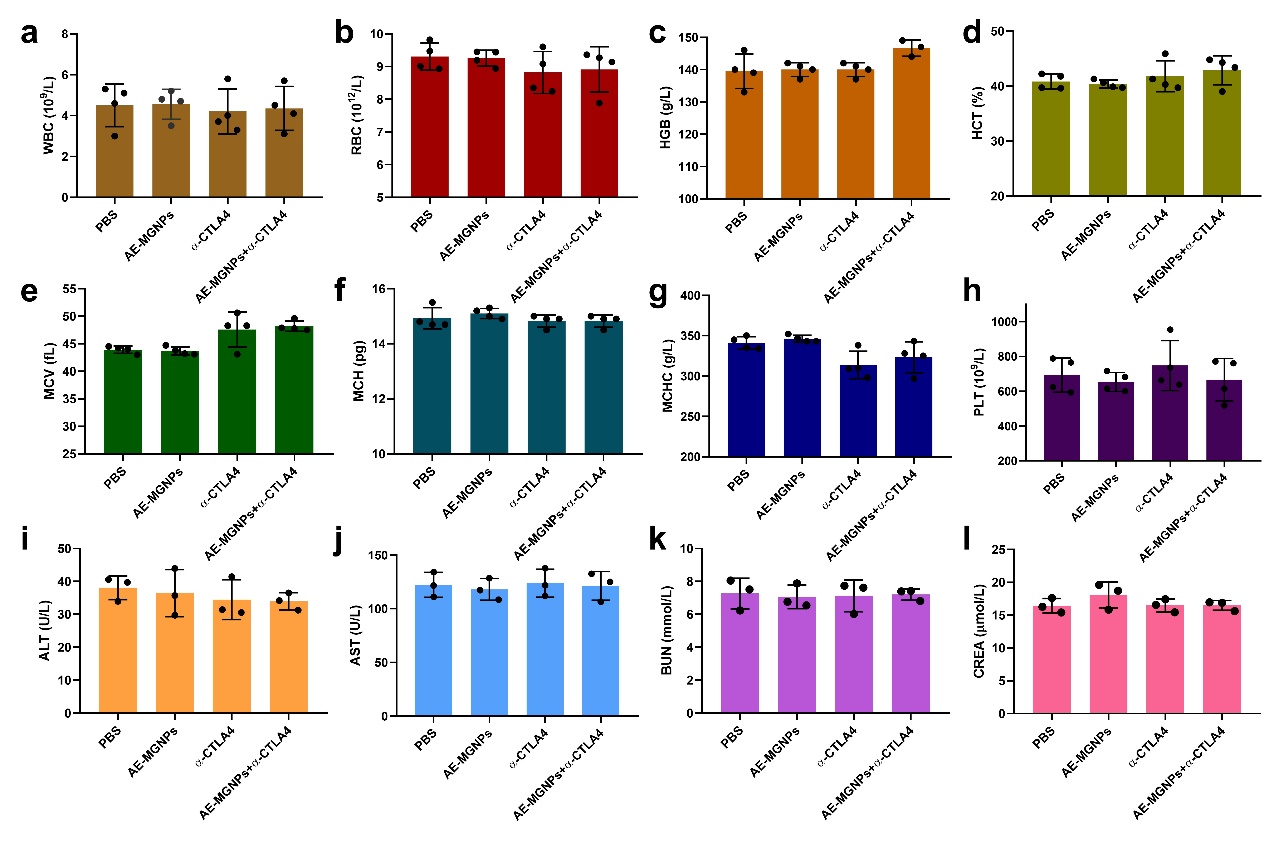


Fig.S16 Hematological tests and serum biochemicals tests of the mice after 23 days of treatment. (A) WBC white blood cells, (B) RBC red blood cells, (C) HGB hemoglobin, (D) HCT hematocrit, (E) MCV mean cell volume, (F) MCH mean corpuscular hemoglobin, (G) MCHC mean corpuscular hemoglobin concentration, (H) PLT blood platelet, (I) ALT alanine transferase, (J) AST aspartate transferase, (K) BUN blood urea nitrogen, (L) CREA creatinine. The data are presented as the mean ± S.D. (n=5)


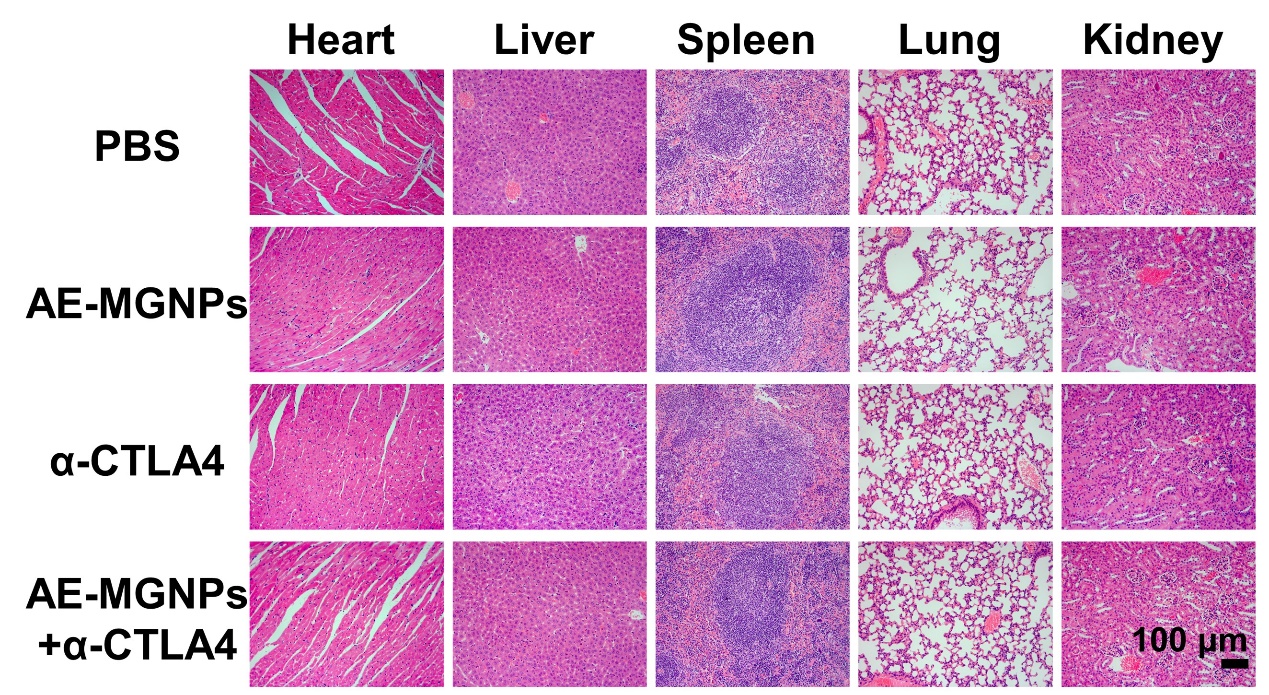


Fig.S17 Histological evaluation of the major organs, including the liver, spleen, kidneys, heart, and lungs, of the CT26 tumor-bearing mice after 23 days of treatment. The scale bars indicate 100 μm.

**Table 1.** Encapsulation efficiency (EE%) and drug loading (DL%) content of different nanoparticle nanoemulsions.

| **Samples** | **EE of**  **GSNO (%)** | **EE of**  **Melittin (%)** | **DL of**  **GSNO (%)** | **DL of**  **Melittin (%)** |
| --- | --- | --- | --- | --- |
| AE-GNPs | 49.73±1.92% | - | 2.37±0.92% | - |
| AE-MNPs | - | 42.93±1.71% | - | 20.48±0.68% |
| AE-MGNPs | 46.03±2.03% | 41.92±2.21% | 2.25±0.87% | 20.1±0.73% |

**Table 2.** Primer sequences used in real-time PCR (Mouse)

| **Name** | **Sequences** |
| --- | --- |
| GAPDH | Forward: 5′-GAAGGTCGGTGTGAACGGAT-3′ |
|  | Reverse: 5′-AATCTCCACTTTGCCACTGC-3′ |
| FAP | Forward: 5′-TTGTTTCGACACCAGCTTTTAG-3′ |
|  | Reverse: 5′-CCACTTGCCACTTGTAATTTGA-3′ |
| α-SMA | Forward: 5′- CCTCATGCCATCATGCGTCT-3′ |
|  | Reverse: 5′- AATCTCACGCTCGGCAGTAG-3′ |
| Sigma-1R | Forward: 5′- GCACTCACATCCACCCAGAAAGG-3′ |
|  | Reverse: 5′- CTTGATCCCAGAGCCCTGAATGC-3′ |

**Table 3.** Primer sequences used in real-time PCR (Human)

| **Name** | **Sequences** |
| --- | --- |
| GAPDH | Forward: 5′-GAGAGACCCTCACTGCTG-3′ |
|  | Reverse: 5′-GATGGTACATGACAAGGTGC-3′ |
| ANGPT1 | Forward: 5′-GATGTCAATGGGGGAGGTT-3′ |
|  | Reverse: 5′-CTCTGACTGGTAATGGCAAAAATA-3′ |
| S1PR1 | Forward: 5′-CAGCAAATCGGACAATTCCT-3′ |
|  | Reverse: 5′-GCCAGCGACCAAGTAAAGAG-3′ |
| ANGPT2 | Forward: 5′- AATAAGCAGCATCAGCCAAC-3′ |
|  | Reverse: 5′- TCAAGTTGGAAGGACCACAT-3′ |
| VEGFA | Forward: 5′- TCTTCAAGCCATCCTGTGTG-3′ |
|  | Reverse: 5′- ATCCGCATAATCTGCATGGT-3′ |
| EGF | Forward: 5′- CTTGTCATGCTGCTCCTCCTG-3′ |
|  | Reverse: 5′- TGCGACTCCTCACATCTCTGC-3′ |
